# Supplementary material for: Post-Diagnostic Statin Use Reduces Mortality in South Korean Patients with Dyslipidemia and Gastrointestinal Cancer
Source: J Clin Med. 2021 May 27;10(11):2361. doi: 10.3390/jcm10112361 (PMC8198926; doi:10.3390/jcm10112361)
Supplement: Supplementary file 1 [file jcm-10-02361-s001.zip › jcm-1234689-supplementary.pdf]

Table S1. Average DDD / 365 for all statins in patients with dyslipidemia.

		Cancer		Non-cancer	
		**Mean**	**SD**	**Mean**	**SD**
Statin	Simvastatin	0.77	± 0.46	0.71	± 0.33
	Lovastatin	0.67	± 0.51	0.71	± 0.46
	fluvastatin	1.67	± 0.31	1.23	± 0.27
	atorvastatin	0.65	± 0.25	0.67	± 0.41
	Pitavastatin	1.02	± 0.30	1.03	± 0.39

Table S2. General characteristics of participants by MPR

	MPR										*p*-value
	non-user		≤25%		≤50%		≤75%		>75%		
**Cancer**											
Yes	184	(26.9)	272	(30.2)	165	(18.3)	162	(18.0)	118	(13.1)	<0.0001
No	16,892	(26.9)	19,484	(31.1)	11,099	(17.7)	9,288	(14.8)	5,964	(9.5)	
**Main treatment medical institutions for dyslipidemia**											
Community health center & Clinic	12,014	(26.7)	14,554	(32.3)	8,242	(18.3)	6,607	(14.7)	3,617	(8.0)	<0.0001
Hospital	1,909	(29.8)	2,447	(38.2)	1,047	(16.4)	646	(10.1)	349	(5.5)	
General hospital	2,324	(25.5)	2,412	(26.5)	1,534	(16.9)	1,496	(16.4)	1,331	(14.6)	
Tertiary hospital	834	(26.9)	343	(11.1)	441	(14.2)	701	(22.6)	785	(25.3)	
**Sex**											
Male	8,772	(24.7)	9,560	(27.0)	4,219	(11.9)	3,341	(9.4)	2,370	(6.7)	<0.0001
Female	8,404	(23.7)	10,196	(28.7)	7,045	(19.9)	6,109	(17.2)	3,712	(10.5)	
**Age**											
30-44	6,320	(44.4)	5,021	(35.3)	1,580	(11.1)	926	(6.5)	382	(2.7)	<0.0001
45-59	7,778	(24.2)	10,338	(32.2)	6,224	(19.4)	4,893	(15.3)	2,850	(8.9)	
60-75	2,978	(17.2)	4,397	(25.4)	3,460	(20.0)	3,631	(21.0)	2,850	(16.5)	
**BMI**											
<18.5	377	(43.9)	224	(26.1)	126	(14.7)	75	(8.7)	56	(6.5)	<0.0001
18.5-22.9	6,237	(30.5)	6,441	(31.5)	3,458	(16.9)	2,700	(13.2)	1,580	(7.7)	
23-24.9	4,625	(25.9)	5,546	(31.1)	3,301	(18.5)	2,709	(15.2)	1,664	(9.3)	
25-29.9	5,288	(23.9)	6,807	(30.8)	3,961	(17.9)	3,572	(16.2)	2,468	(11.2)	
≥30	549	(22.8)	738	(30.6)	418	(17.3)	394	(16.3)	314	(13.0)	
**Residual area**											
Capital area	7,700	(27.9)	7,685	(27.9)	4,708	(17.1)	4,431	(16.1)	3,065	(11.1)	<0.0001

Metropolitan	4,374	(25.3)	5,768	(33.4)	3,305	(19.1)	2,422	(14.0)	1,397	(8.1)	
Other	5,002	(26.6)	6,303	(33.6)	3,251	(17.3)	2,597	(13.8)	1,620	(8.6)	
**Income**											
Low	3,727	(25.3)	4,684	(31.8)	2,676	(18.2)	2,231	(15.2)	1,402	(9.5)	<0.0001
Low-moderate	4,222	(27.3)	5,013	(32.4)	2,673	(17.3)	2,262	(14.6)	1,297	(8.4)	
Moderate-high	3,952	(27.9)	4,383	(30.9)	2,473	(17.4)	2,034	(14.3)	1,347	(9.5)	
High	5,175	(26.9)	5,676	(29.5)	3,442	(17.9)	2,923	(15.2)	2,036	(10.6)	
**Year of diagnosis**											
2002-2005	2,749	(21.9)	3,288	(26.2)	2,489	(19.8)	2,464	(19.6)	1,575	(12.5)	<0.0001
2006-2010	5,531	(23.9)	6,980	(30.2)	4,323	(18.7)	3,720	(16.1)	2,554	(11.1)	
2011-2015	8,796	(31.5)	9,488	(33.9)	4,452	(15.9)	3,266	(11.7)	1,953	(7.0)	
